# Low Health Literacy and Evaluation of Online Health Information: A Systematic Review of the Literature

**DOI:** 10.2196/jmir.4018

**Published:** 2015-05-07

**Authors:** Nicola Diviani, Bas van den Putte, Stefano Giani, Julia CM van Weert

**Affiliations:** ^1^Amsterdam School of Communication Research / ASCoRDepartment of Communication ScienceUniversity of AmsterdamAmsterdamNetherlands; ^2^Trimbos InstituteNetherlands Institute for Mental Health and AddictionUtrechtNetherlands; ^3^University LibraryUniversity of AmsterdamAmsterdamNetherlands

**Keywords:** health information seeking, online health information, information quality, health literacy

## Abstract

**Background:**

Recent years have witnessed a dramatic increase in consumer online health information seeking. The quality of online health information, however, remains questionable. The issue of information evaluation has become a hot topic, leading to the development of guidelines and checklists to design high-quality online health information. However, little attention has been devoted to how consumers, in particular people with low health literacy, evaluate online health information.

**Objective:**

The main aim of this study was to review existing evidence on the association between low health literacy and (1) people’s ability to evaluate online health information, (2) perceived quality of online health information, (3) trust in online health information, and (4) use of evaluation criteria for online health information.

**Methods:**

Five academic databases (MEDLINE, PsycINFO, Web of Science, CINAHL, and Communication and Mass-media Complete) were systematically searched. We included peer-reviewed publications investigating differences in the evaluation of online information between people with different health literacy levels.

**Results:**

After abstract and full-text screening, 38 articles were included in the review. Only four studies investigated the specific role of low health literacy in the evaluation of online health information. The other studies examined the association between educational level or other skills-based proxies for health literacy, such as general literacy, and outcomes. Results indicate that low health literacy (and related skills) are negatively related to the ability to evaluate online health information and trust in online health information. Evidence on the association with perceived quality of online health information and use of evaluation criteria is inconclusive.

**Conclusions:**

The findings indicate that low health literacy (and related skills) play a role in the evaluation of online health information. This topic is therefore worth more scholarly attention. Based on the results of this review, future research in this field should (1) specifically focus on health literacy, (2) devote more attention to the identification of the different criteria people use to evaluate online health information, (3) develop shared definitions and measures for the most commonly used outcomes in the field of evaluation of online health information, and (4) assess the relationship between the different evaluative dimensions and the role played by health literacy in shaping their interplay.

##  Introduction

Recent years have witnessed a dramatic increase in consumer online health information seeking [1]. Online health information deserves particular attention because studies on the content of health-related websites have highlighted inaccuracies that raise concerns about the quality of the online health information encountered by consumers [2-6]. The limited accuracy of information is often the result of one of the distinctive features of the Internet, that is, that anyone can potentially publish health-related information. Online health information seeking thus poses several major challenges to health information users, as it requires them to undertake an active role in evaluating a vast amount of often unverified health information on the Internet [7]. As a result, people experiencing difficulties evaluating online health information may be exposed to wrong or incomplete information, which has been shown to be related to adverse health outcomes, such as low participation in screening programs or low adherence to treatments [8]. Evidently, more attention needs to be given to the issue of quality of online health information and in particular to people’s ability to evaluate it [2-6].

Several guidelines and checklists to improve the quality of online health information have been developed, for example by the Standford Persuasive Tech Lab, the Health On the Net Foundation (HONcode), Web Médica Acreditada, and Centrale santé (Netscoring criteria) (see Kim et al [9] for a summary view). These tools can be useful for Web designers and providers of health information to develop high-quality health websites. At the same time, the guidelines could be used by users as evaluation criteria to assess online health information. However, these criteria are likely known and adopted only by specific segments of the population, resulting in disparities in people’s ability to evaluate online health information. The knowledge gap hypothesis, for instance, states that as a result of increasing mass media exposure, individuals in the higher socioeconomic strata of society tend to acquire information faster than people in lower ones. So the gap in knowledge between the two tends to increase rather than decrease [10]. It is likely that traditionally disadvantaged groups—such as those with lower education or lower health literacy—will be the ones at higher risk for disparities in this context [11].

Among the determinants of health disparities, people’s health literacy has been proven to play a crucial role in the context of health information seeking. Health literacy has originally been defined as “the cognitive and social skills which determine the motivation and ability of individuals to gain access, to understand, and use information in ways which promote and maintain good health” [12]. A closer look at the different conceptualizations of health literacy proposed in the last years (eg, [12-19]) shows that almost all of them explicitly or implicitly include people’s ability to deal with (ie, obtain, process, evaluate, and use) health information among the skills a person should possess in order to be considered health literate. Several studies have provided evidence of differences in how people with different levels of health literacy seek, find, understand, and use online health information. For instance, low health literate people have been shown to search less for health information, choose different information sources, and have a poorer ability to interpret medication labels or health messages [20-23]. In contrast, little attention has been devoted to how consumers—and in particular those with low health literacy—evaluate online health information [24]. Additionally, to date, no studies have systematically summarized existing evidence on the role of health literacy in the context of the evaluation of online health information.

The main objective of this study was to address this research gap and provide a comprehensive description of how low health literacy impacts people’s evaluation of online health information. This translates to four distinct research questions aimed at understanding whether and how people’s health literacy is related to their ability to evaluate online health information (RQ1), perceived quality of online health information (RQ2), people’s trust in the Internet as a source of online health information (RQ3), and the use of evaluation criteria for online health information (RQ4).

The rationale behind RQ1 is the fact that a relationship between health literacy and ability to evaluate the quality of health information has been explicitly or implicitly suggested by several health literacy conceptualizations [12-19] but has not been systematically verified so far.

Information evaluation, however, does not depend only on the characteristics of the audience (in our case, people’s ability to evaluate online health information). As acknowledged already at the very beginning of scholarly interest in the field of credibility, characteristics of the message and the source can play a role as well [25-28]. This is the reason why RQ2 is about the relationship between health literacy and perceived quality of online health information (message level). Perceived information quality is a multifaceted concept encompassing several dimensions, which in turn can be grouped in different categories [29,30]. For the purposes of this review, we will focus on the dimensions of perceived reliability and accuracy, which Wang and Strong [31] have defined as intrinsic information quality.

It has been found that perceived information quality does not necessarily imply intention to rely on it [32]. Someone could, for instance, perceive a message as being of high quality but not trust the source because of external factors (such as previous negative experiences) and thus decide not to act on the information. Therefore, RQ3 concerns overall trust in the Internet as a source of health information. In the context of computer-mediated communication, trust has been defined in terms of dependability and is a subjective judgment about whether a person (or, in the case of online health information, a digital object) is worth being relied on [32,33].

Last, as past research in other fields has shown that people use several different criteria when evaluating online information, including relying on formal (eg, color of the webpage) or contextual (eg, position in Google search results) aspects of the website, or evaluating the information based on previous knowledge [34], RQ4 is aimed at understanding whether health literacy plays a role in the choice and use of these evaluation criteria.

Answers to these four questions will provide us with a new and more comprehensive understanding of the role played by low health literacy in the evaluation of online information. At the same time, the results of the review will allow us to identify possible areas where consumer education could have an impact on improving low health literate people’s ability to correctly evaluate online health information.

## Methods

### Data Sources and Search Strategy

During the third week of January 2014, five academic databases from different relevant disciplines (Medline, PsycINFO, CINAHL, Web of Science, and Communication and Mass Media Complete) were systematically searched for peer-reviewed literature describing consumers’ evaluation of online health information. No time limits were set because the topic of online health information seeking is relatively recent. Search terms used included a combination of Medical Subject Headings (MeSH) terms and free terms covering the four domains, “online information”, “health”, “evaluation”, and “health literacy”, combined with the Boolean operator AND (see Multimedia Appendix 1). A preliminary search showed that the number of articles explicitly mentioning health literacy was limited, so the search was modified to include some of its most common proxies or indicators. Since health literacy has been defined as a set of skills [12], only skills-based indicators (eg, educational attainment, reading ability, or general literacy) were included in the search. These indicators can be considered as proxies of health literacy because of their conceptual similarities (eg, they all refer to teachable skills) and the existence of a direct link between the concepts. Other common, mainly sociodemographic, indicators (eg, age, income, or ethnicity) were excluded because of the more complex nature of their relationship with health literacy. The original search strategy was developed for PsycINFO and subsequently adapted to the peculiarities and requirements of the other databases. These terms were included in the original search, allowing us to refine it. References cited in included articles were reviewed manually, and a Google Scholar and Web of Science search for recent articles citing the ones included in the review was performed in order to identify further additional articles relevant to this review (snowball method).

### Study Inclusion/Exclusion Criteria

A two-phase screening process was conducted. After de-duplication, the first author (ND) screened titles and abstracts of all retrieved articles in order to identify possible relevant articles (first phase). Abstracts were selected for full text screening (second phase) if they (1) were written in English, (2) reported original results, qualitative or quantitative, (3) studied consumer online health information, (4) mentioned evaluation of the information by consumers/patients, and (5) had been conducted in a low health literacy population (or in a sample of the above-described proxies of low health literacy) OR subgroup analyses were conducted in a sample of low health literate people (or proxies of low health literacy). Excluded were non-empirical articles (such as reviews, commentaries, or editorials), articles describing empirical studies conducted among health care providers, content analyses of websites, quality assessments of websites, and articles reporting on research conducted in samples that were not explicitly described as low health literate (or proxies) and did not present subgroup analyses for the low health literacy (or proxies) group. No selection based on the country where the study was conducted was made, and the same inclusion criteria were used for each country. In order to estimate reliability of the screening process, 10% of the abstracts and all the selected full texts were independently assessed by a second researcher. Initial intercoder agreement (Cohen’s kappa >.70 for both title/abstract and full-text screening) was substantial [35], and all disagreements regarding full texts were resolved during consensus meetings that were held on a regular basis during the whole screening process.

### Data Extraction

#### Overview

Besides the formal characteristics of the included papers—author(s), publication date, study design, study population, and sampling*—*data were extracted from all articles on the following aspects, which were deemed relevant to answer our research questions.

#### Predictors

Data were extracted about the predictors used in the study and the measures used to assess them. The main predictor of interest was health literacy, which can be assessed using different tools, for example, the Rapid Estimate of Adult Literacy in Medicine (REALM) [36], the Short Test of Functional Health Literacy in Adults (S-TOFHLA) [37,38], or the Newest Vital Signs (NVS) [39]. As mentioned earlier, however, included studies could also describe differences in one of the outcomes of interest related to differences in education or other skills-based proxies for health literacy [40]. Each study could address one or more predictors.

#### Outcomes

Data were extracted on the outcomes addressed in the studies and the measures used to assess them. The four outcomes of interest were (1) ability to evaluate online health information, (2) perceived quality of online health information, (3) trust in the Internet as a source of online health information, and (4) use of evaluation criteria for online health information. Each study could address one or more outcomes.

#### Association Between Predictors and Outcomes

All qualitative or quantitative evidence of (or lack of) an association between one of the predictors and one of the four outcomes of interest were extracted.

### Data Synthesis

Given the heterogeneity of study designs, samples, predictors, and outcome measures in our articles pool, results could not be synthetized quantitatively using meta-analytic techniques. The findings were therefore synthetized narratively and structured according to the different outcomes under investigation.

## Results

### Included Studies

**Figure 1:**
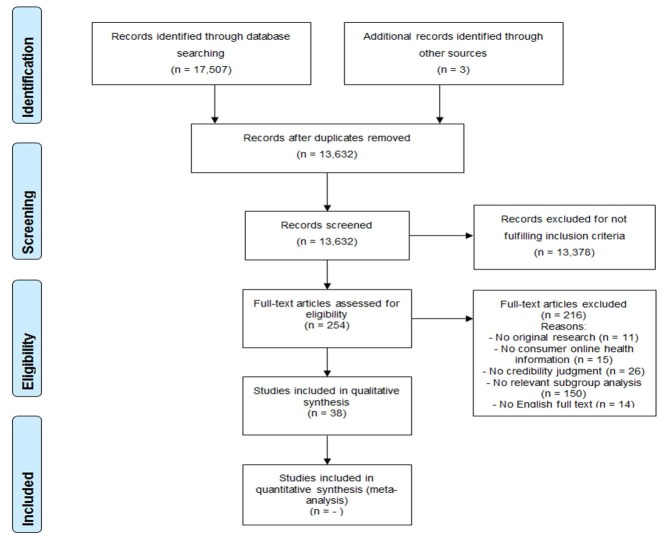
Flow diagram of the screening process.

The initial search resulted in 17,507 articles. In the process of reviewing articles identified through the initial searches, 3 additional articles were identified through cited references, bringing the total to 17,510 articles. After duplicates were removed, the remaining number of articles was 13,632, of which 13,378 were discarded after reviewing titles and abstract. An additional 216 articles were discarded after reviewing the articles in their entirety, resulting in 38 articles [24,41-77]. The whole process is illustrated in detail by the flow diagram in Figure 1.

### Characteristics of Included Studies

The basic characteristics of the final pool of articles included in the systematic review are described in Table 1 [41-77]. The 38 studies were published between 2001 and 2013. Most of them were conducted in North America (24/38; 63%), five were conducted in Europe (13%), four (11%) in Asia, four (11%) in Australia, and one in Africa (3%). Study populations varied widely, ranging from the general population to specific patient groups, and so did sample sizes, ranging from N=8 up to N=8586. All studies were non-experimental, with the vast majority being cross-sectional surveys (35/38; 92%), and the remaining were qualitative studies (1 focus group study [60] and 2 qualitative observational studies [45,50]).

According to commonly used approaches for rating the quality of evidence in systematic reviews (eg, Grades of Recommendation, Assessment, Development and Evaluation [GRADE]; see [78]), the quality level of the evidence has to be considered low because all the studies included in this review are non-interventional in nature.

**Table 1:** Characteristics of included studies.

Author(s), date	Country	Study type	Sample	Sample size, N
AlGahmdi & Moussa, 2012 [41]	Saudi Arabia	Cross-sectional	Random sample of male and female outpatients and visitors attending a public University Hospital in Riyadh, Saudi Arabia	801
Bates et al, 2007 [42]	United States	Cross-sectional	Community-wide convenience sample through intercept survey methods. Participants were recruited at high-traffic areas in a regional hub city in southeastern Ohio	519
Benotsch et al, 2004 [43]	United States	Cross-sectional	Individuals with human immunodeficiency virus recruited from neighborhoods in inner city Atlanta, Georgia	324
Bernhardt et al, 2004 [44]	United States	Cross-sectional	In-person surveys administered to diverse respondents in four different locations in two states, including a small and large city in the Southeastern United States, and a small and large city in the Northeastern United States. Online surveys were administered on a webpage that was promoted to diverse respondents using emails and word-of-mouth	858
Birru et al, 2004 [45]	United States	Observational study	Subjects enrolled in a reading assistance program at Bidwell Training Center in Pittsburgh, PA	8
Borzekowski & Rickert, 2001 [46]	United States	Cross-sectional	Sample of 10th grade students from a diverse community near NY	412
Clayman et al, 2010 [47]	United States	Cross-sectional	Hispanics-Latinos of the 2005 Health Information National Trends Survey (HINTS) sample	496
Dart, 2008 [48]	Australia	Cross-sectional	Three different Australian communities: low socioeconomic sample, mid-high socioeconomic sample, and university sample	714
Dutta-Bergman, 2003 [49]	United States	Cross-sectional	Stratified random sample of approximately US adults (Porter Novelli HealthStyles database)	2636
Feufel & Stahl, 2012 [50]	Germany	Observational study	Native German-speaking adults	22
Gauld & Williams, 2009 [51]	Australia & New Zealand	Cross-sectional	Non-representative sample of Australians and New Zealanders	406
Ghaddar et al, 2012 [52]	United States	Cross-sectional	Random sample of high school students in South Texas	261
Helft et al, 2005 [53]	United States	Cross-sectional	Convenience sample of patients from the WMH Oncology Specialty Outpatient Clinic, Indianapolis	200
Hesse et al, 2005 [54]	United States	Cross-sectional	Nationally representative sample of US adults 18+ (HINTS 2002-03)	6369
Ishikawa et al, 2012 [55]	Japan	Cross-sectional	Nationally representative sample of people aged 15-75 years in Japan	1311
Kalichman et al, 2006 [56]	United States	Cross-sectional	HIV-positive men and women who use the Internet recruited from AIDS service organizations, health care providers, social service agencies, and infectious disease clinics in inner-city areas of Atlanta, GA	419
Knapp et al, 2011a [57]	United States	Cross-sectional	Parents whose children with special health care needs were enrolled in Florida’s Medicaid and State Children’s Health Insurance Plan (SCHIP)	2371
Knapp et al, 2011b [58]	United States	Cross-sectional	Parents whose children are in a pediatric palliative care program in Florida	129
Lawson et al, 2011 [59]	New Zealand	Cross-sectional	Sample of New Zealanders drawn from the electoral roll	8291
Mackert et al, 2009 [60]	United States	Focus groups	Parents from a midsized city in the southwestern United States 18 years of age or older, at or below median income for the area, who had not completed a 4-year college degree nor worked in the health care field	43
Maguire et al, 2011 [61]	Australia	Cross-sectional	Australian adults with schizophrenia (recruited from both community and inpatient settings) and general practice attendees	301
Maraziene et al, 2012 [63]	Lithuania	Cross-sectional	Randomly selected sample of Lithuanian citizens	1763
Marrie et al, 2013 [63]	United States	Cross-sectional	US people with multiple sclerosis enrolled (voluntary) in the Consortium of MS Centers developed the North American Research Committee on Multiple Sclerosis (NARCOMS) Registry	8586
Murray et al, 2003 [64]	United States	Cross-sectional	Household probability sample of US adults (18+) from the 48 contiguous states	3209
Neter & Brainin, 2012 [65]	Israel	Cross-sectional	Adult (18+) Israeli population	4286
Nguyen & Bellamy, 2006 [66]	United States	Cross-sectional	Non-Hispanic Asians and non-Hispanic whites form the 2003 HINTS	4395
Nwagwu, 2007 [67]	Nigeria	Cross-sectional	In-school, and out-of-school adolescents in Owerri, Nigeria	1145
Oh et al, 2012 [68]	United States	Cross-sectional	Korean Americans ≥40 years	254
Richter et al, 2009 [69]	Germany	Cross-sectional	Patients with inflammatory rheumatic diseases (rheumatoid arthritis, systemic Lupus erythematosus (SLE), spondyloarthritis (SpA) regularly scheduled for a visit in our Rheumatology outpatient clinic at the University Clinic Düsseldorf	153
Smith, 2011 [70]	United States	Cross-sectional	Nationally representative sample of adults in the United States from the 2008 Annenberg National Health Communication Survey (ANHCS)	3656
Soederberg Miller & Bell, 2012 [71]	United States	Cross-sectional	Nationally representative sample of US adults from the Health Information National Trends Survey (HINTS)	3796
Van der Vaart et al, 2011 [72]	The Netherlands	Cross-sectional	Sample of patients with rheumatic diseases (Study 1) and stratified sample of the Dutch population (Study 2)	277
van Deursen & van Dijk, 2011 [73]	The Netherlands	Cross-sectional	Stratified random sample of adults (18+) living in the region of Twente, The Netherlands	88
Yan, 2010 [74]	Hong Kong & Kowloon	Cross-sectional	Convenience sample recruited in urban public areas including shopping locations (in Hong Kong Island and Kowloon) and subway stations	443
Ye, 2011 [24]	United States	Cross-sectional	Nationally representative sample of US adults (HINTS)	7674
Zhao, 2010 [75]	United States	Cross-sectional	2005 HINTS sample (foreign-born and US born)	5393
Zoellner et al, 2009 [76]	United States	Cross-sectional	Proportional quota sample of adult residents in the Mississippi Delta region.	177
Zulman et al, 2011 [77]	United States	Cross-sectional	Adults 50 years of age and older in the United States	1450

### Predictors Included in the Studies

Only four of the included studies (4/38, 11%) specifically described the relationship between health literacy and one or more of the four outcomes of interest [45,52,60,76]. Health literacy was measured using different instruments: the NVS [52,76], the TOFHLA [43], and S-TOFHLA [60].

The majority of the included studies (33/38, 87%) described the relationship between educational level and one or more of the outcomes. In most of them, educational level was operationalized either as number of years of education or as the highest achieved degree. In some cases, however, other operational definitions were used. One study compared a university sample with a mid-high socioeconomic and a low socioeconomic sample [48], two compared different ethnic groups with different educational levels [66,75], one compared in-school versus out-of-school young people [67], and one last study compared students in different grades and enrolled in programs with or without a health focus within the same school [52].

Five studies (13%) described the relationship between other skills-based proxies for health literacy and one or more of the outcomes. These skills included reading comprehension [43], comfort speaking English [47], general literacy [45], and ability to understand health information [24]. Although it could be argued that this last skill is conceptually very similar to health literacy, the authors did not define it as such in their paper. In addition, health literacy was not measured with a recognized measure but by a single item. In the fifth study, a distinction was made between skilled (younger than 30 years, higher educational level and more experienced using the Web) and less-skilled (50 or older) participants [50]. As some studies reported results related to more than one predictor, percentages add up to more than 100%, and studies might be referred to more than once throughout the results.

### Outcomes

#### Overview

The majority of the studies included in the review included trust in online health information as an outcome variable (53%, 20/38). The second most common outcome was evaluation ability, included in 26% of the studies (10/38), followed by perceived quality of online health information (defined in the studies as perceived credibility, reliability, accuracy, and worth), which was included in 21% of the studies (8/38). The least frequent outcome variable in the pool of articles was the use of evaluation criteria, which was included in 5 studies only (14%). As some studies reported results on more than one outcome, percentages add up to more than 100%, and studies might be referred to more than once throughout the results.

#### Ability to Evaluate Online Health Information

Our first research question focused on the relationship between health literacy and ability to evaluate online health information. This specific aspect was addressed in 10 (26%) of the 38 studies (see Table 2 [43,45,52,56-58,64,65,72,73]). Evaluation ability was mostly assessed using self-report measures. Five studies [52,57,58,65,72] used the eHealth Literacy scale (eHEALS)—a measure assessing several aspects related to online health information seeking, including people’s perceived ability to evaluate online health resources and to distinguish high quality from low quality online health information—or some of its items [79]. Other studies asked the respondents to self-assess their ability to evaluate online health information by means of a single item measure [64] or qualitatively by means of an open question [45]. Among the studies using objective measures, van Deursen and van Dijk [73] asked their participants to perform an evaluation task derived from the eHEALS. In the other studies, the participants were asked to evaluate websites of varying quality [43,56].

Two studies assessed the role of health literacy in people’s ability to evaluate online health information. The first study showed that low health literacy was associated with lower eHEALS scores [52], while in the second study low health literacy was shown to be associated with lower quality ratings of a high-quality website and higher quality ratings of a low-quality website [43].

Six out of the nine studies focusing on educational level showed a positive relationship between educational attainment and perceived or actual ability to evaluate online health information [43,52,57,58,65,73]. The studies by Murray et al [64] and van der Vaart et al [72] did not find any significant difference among different education groups. A last study found that lower education was associated with assigning a higher quality rating to a low-quality website but did not find any association between education and evaluation of a high-quality website [56].

Last, two studies reported on ability to evaluate online health information in relation to other skills. In a small qualitative study conducted in a sample with low general literacy, 7 out of the 8 respondents reported finding it very easy to locate trustworthy health information on the Internet [45]. The second study identified a positive association between reading ability and correct evaluation of the quality of a high-quality and a low-quality website [43].

#### Perceived Quality of Online Health Information

Eight studies (21%) reported on perceived quality of online health information. Except in one case where a multidimensional scale was used [46], this aspect was measured by means of single-item measures. Most studies did not refer directly to information quality but to one of its dimensions, that is, reliability [51,69,74], perceived accuracy [44,53,67], and perceived worth [46] (see Table 3 [44,46,50,51,53,67,69,74]). No studies on perceived information quality included health literacy as a predictor.

The majority of the studies focusing on educational level failed to find significant associations between education and perceived quality of online health information. One study found a positive association [53], and one a negative association [44]. Another study found contrasting results: while out-of-school adolescents tended to describe online health information as more accurate than in-school adolescents, the latter group assigned higher overall quality to online health information as compared to out-of-school adolescents [67].

Only one study reported on the relationship between skills-based proxies for health literacy and perceived quality of online health information. The study, however, did not find any differences among skilled and less-skilled participants: both groups doubted the quality of online health information [50].

**Table 2:** Outcome 1: Ability to evaluate online health information.

Author(s), date	Predictor	Specific measure used	Result
Benotsch et al, 2004^a^[43]	Health literacy (TOFHLA)	Quality rating of health information from reputable (JAMA) and unfounded (AIDS cure) webpages (5 dimensions: accuracy, amount of detail, trustworthiness/credibility, relevance, and usefulness)	Lower health literacy scores predict higher quality ratings for the AIDS cure webpage (unfounded) and lower quality ratings for the JAMA webpage (reputable) (*P*<.01).
Ghaddar et al, 2012^b^[52]	Health literacy (NVS)	eHEALS^c^	Students identified as possibly or likely low health literate present significantly lower eHEALS scores than those with adequate health literacy (*P*<.05).
Benotsch et al, 2004^a^[43]	Educational level	Quality rating of health information from reputable (JAMA) and unfounded (AIDS cure) webpages (5 dimensions: accuracy, amount of detail, trustworthiness/credibility, relevance, and usefulness)	Individuals with fewer years of education assign more credibility to unfounded information (*P*<.01). Educational level is unrelated to perceived quality of the JAMA webpage.
Ghaddar et al, 2012^b^[52]	Educational level (different grade levels; health classes)	eHEALS^c^	Freshmen and sophomore students and those who have not taken a health course have lower eHEALS scores relative to students in higher grade levels and those enrolled in a health course (*P*<.001). eHEALS scores are significantly lower among students from the non-medical focused campuses compared to the 2 high schools with a focus on medical education (*P*<.001).
Kalichman et al, 2006 [56]	Educational level	Quality rating of health information from reputable (JAMA) and unfounded (AIDS cure) web pages	Less education predicts assigning higher credibility to unfounded Internet information (*P*<.001). Education does not have an impact on the evaluation of the reputable webpage.
Knapp et al, 2011a[57]	Educational level	eHEALS (Item 6: “I have the skills I need to evaluate the health resources I find on the Internet” and Item 7: “I can tell high-quality health resources from low-quality health resources on the Internet”)	Parents without college education feel less confident in having the skills to evaluate the health resources they find on the Internet (*P*<.05) and feel less able to tell high-quality health resources from low-quality health resources on the Internet (*P*<.001) compared to those with college education.
Knapp et al, 2011b[58]	Educational level	eHEALS^c^	Not having a high school diploma is associated with a 2.5-point decrease in overall eHealth literacy (*P*<.05).
Murray et al, 2003 [64]	Educational level	Perceived ability to appraise online health information	No significant effect of education on self-rated ability in appraising online health information.
Neter & Brainin, 2012 [65]	Educational level	eHEALS^c^	Lower education is associated with lower eHealth literacy (*F*_1,1274_=5.43, *P*<.02).
Van der Vaart et al, 2011 [72]	Educational level	eHEALS^c^	No significant correlation between educational level and eHEALS scores.
Van Deursen & Van Dijk, 2011 [73]	Educational level	Number of information tasks^d^ (derived from the eHEALS) completed successfully	Educational level is positively correlated with the number of information tasks completed successfully (β=.56, *P*<.001).
Benotsch et al, 2004^a^[43]	Other skills-based proxies for health literacy – Reading comprehension	Quality rating of health information from reputable (JAMA) and unfounded (AIDS cure) webpages (5 dimensions: accuracy, amount of detail, trustworthiness/credibility, relevance, and usefulness)	Poorer reading comprehension predicts higher quality ratings for the AIDS cure webpage, whereas higher reading comprehension predicts higher quality ratings for the JAMA webpage (*P*<.01).
Birru et al, 2004 [45]	Other skills-based proxies for health literacy – Low general literacy (3rd to 8th grade level) only sample	Perceived ability to locate trustworthy online health information	7 out of 8 subjects report that they find it very easy to locate trustworthy information on the Internet. The eighth subject notes that it is moderately easy to find information that is trustworthy on the Internet.

^a^Study reported three times because it described the impact of health literacy, educational level, and other skills-based proxies for health literacy on the ability to evaluate the credibility of online health information.

^b^Study reported twice because it described the impact of both health literacy and educational level on the ability to evaluate the credibility of online health information.

^c^The eHEALS (eHealth Literacy scale) includes specific items about people’s perceived ability to evaluate the quality of online health information (Item 6: “I have the skills I need to evaluate the health resources I find on the Internet” and Item 7: “I can tell high-quality health resources from low-quality health resources on the Internet”). Specific data for these items are, however, not presented in the paper.

^d^Information tasks included choosing a website or a search system to seek information, defining search options or queries, selecting information on websites or in search results, and evaluating information sources. Specific data for the task “Evaluating information sources” are not presented in the paper.

**Table 3:** Outcome 2: Perceived quality of online health information.

Author(s), date	Predictor	Specific measure used	Result
Bernhardt et al, 2004 [44]	Educational level	Perceived accuracy of online health information	Less educated respondents perceive online health information to be more accurate (*P*<.05).
Borzekowski & Rickert, 2001 [46]	Educational level	Composite assessing perceived worth, trustworthiness, use, and relevance of online health information	No significant effect of educational level on the outcome.
Gauld & Williams, 2009 [51]	Educational level	Perceived reliability of online health information	Educational level is not correlated to perceived reliability of online health information.
Helft et al, 2005 [53]	Educational level	Perceived accuracy of online health information	Less educated patients are less likely to believe that online health information is accurate (*r*=.0417; *P*<.05).
Nwagwu, 2007 [67]	Educational level – In-school vs Out-of-school	Perceived accuracy and quality of online health information	The out-of-school groups describes more often the information as accurate. Overall, however, the in-school group assess online health information to be of higher quality more often than the out-of-school.
Richter et al, 2009 [69]	Educational level	Perceived reliability of online information	No significant effect of education on perceived reliability of online information.
Yan, 2010 [74]	Educational level	Perceived reliability of online health information	No significant effect of educational level on perceived reliability of online health information.
Feufel & Stahl, 2012 [50]	Other skills-based proxies for health literacy – Skilled (˂30 years of age, had a higher level of education, and were more experienced using the Web) vs less-skilled (≥50 years of age)	Attitudes towards the quality of online health information	Health information seekers in both cohorts doubt the quality of information retrieved online; among poorly skilled seekers, this is mainly because they doubt their skills to navigate vast amounts of information; once a website is accessed, quality concerns disappear in both cohorts.

#### Trust in Online Health Information

Trust in online health information was the outcome measure of more than half of the studies included in this review (20/38, 53%) (Table 4 [24,41,47,48,54,55,59,61,63,66-71,74-76]). Among those, three studies asked the participants to rate their trust in online health information on specific health topics (cancer and nutrition [54,66,76]). All other studies referred to general trust in online health information and assessed it by means of a single-item measure.

Only one study investigated trust in online health information in relation to health literacy and did not find any significant relationship [76].

Ten out of the 17 studies reporting on the relationship between educational level and trust in online health information identified a positive association. Among the other studies, two found a negative association [59,66] and four found no significant association [24,48,69,74] between educational level and trust. One study reported contrasting results: Maguire et al [61] found a positive association between education and trust among respondents with schizophrenia but not among non-schizophrenia respondents.

Two studies reported on the relationship between skills-based proxies for health literacy and trust in online health information. A positive association between comfort speaking English [47] and ease in understanding health information [24] and trust was found.

**Table 4:** Outcome 3: Trust in online health information.

Author(s), date	Predictor	Specific measure used	Result
Zoellner et al, 2009 [76]	Health literacy (NVS)	Trust in food, diet, or nutrition-related online health information	No significant effect of health literacy on trust in online health information.
AlGahmdi & Moussa, 2012 [41]	Educational level	Trust in online health information	Fewer individuals with lower education always trust online health information (*P*<.001).
Dart, 2008 [48]	Low socioeconomic (LSE) vs mid-high (MSE) socioeconomic vs university	Trust in online health information	Most respondents in all three groups (LSE, 58.4%; MSE, 63.7%; university, 64.5%) are unsure of the trustworthiness or distrusted online health information (no significance level reported).
Hesse et al, 2005 [54]	Educational level	Trust in cancer-related online health information	Education is positively associated with trust in cancer-related online information (*P*<.01).
Ishikawa et al, 2012 [55]	Educational level	Trust in online health information	Participants with high school education or less report less trust in online health information than those with higher education (OR 0.68, 95% CI 0.51-0.92).
Lawson et al, 2011 [59]	Educational level	Trust in health information from media (including the Internet)	Education is negatively associated with trust in health information from media (no statistics reported).
Maguire et al, 2011 [61]	Educational level	Trust in online health information	A lower level of education makes it more than twice less likely that a person with schizophrenia would trust online health information (OR 2.24, *P*<.01). There are no education-related differences among respondents without schizophrenia.
Maraziene et al, 2012 [63]	Educational level	Trust in online health information	People with lower education tend to trust the Internet less than their better educated counterparts (*P*<.05).
Marrie et al, 2013 [63]	Educational level	Trust in online health information	Respondents with a high school degree or less are less likely to have some/a lot of trust in online health information compared to those with an associate’s degree (OR 1.31, 95% CI 1.10-1.57), bachelor’s degree (OR 1.37, 95% CI 1.17-1.61), and graduate degree (OR 1.30, 95% CI 1.10-1.55).
Nguyen & Bellamy, 2006 [66]	Whites vs Asians (significantly different educational background)	Trust in cancer-related online health information	Asians (lower educational level) are more likely to trust cancer-related online information than whites (OR 0.54, *P*<.05).
Nwagwu, 2007 [67]	In-school vs Out-of-school	Trust in online health information	Out-of-school respondents report the information as trustworthy less often than the in-schools (no statistics reported).
Oh et al, 2012 [68]	Educational level	Trust in online health information	Respondents with 12 or fewer years of education are 3.1 times less likely to trust online health information a lot than were those with more than 12 years (95% CI 1.1-8.6).
Richter et al, 2009 [69]	Educational level	Confidence in online health information	No significant effect of education on confidence in online health information.
Smith, 2011 [70]	Educational level	Trust in online health information	Education is positively associated with trust in online health information (*P*<.001).
Soederberg Miller & Bell, 2012 [71]	Educational level	Trust in online health information	Education is significantly correlated with trust in online health information (*P*<.01)
Yan, 2010 [74]	Educational level	Confidence in online health information	No significant effect of educational level on confidence in online health information.
Ye, 2011^a^ [24]	Educational level	Trust in online health information	Educational level is not correlated to trust in online health information.
Zhao, 2010 [75]	US-born vs Foreign-born (significantly different educational background)	Trust in online health information	Foreign-born Hispanics have lower trust in online health information compared with their US-born counterparts (higher educational level) (55% vs 86%, *P*=.016).
Zulman et al, 2011 [77]	Educational level	Trust in online health information	Those with high school or less are significantly less likely to trust online health information than college graduates (OR 2.47, *P*<.001).
Clayman et al, 2010 [47]	Other skills-based proxies for health literacy – Comfort speaking English	Trust in online health information	Those less comfortable speaking English report lower trust in online health information compared with those more comfortable speaking English (*P*<.01).
Ye, 2011^a^ [24]	Other skills-based proxies for health literacy – Hard to understand health information	Trust in online health information	The harder the health information is to understand, the less trust there is in online health information, *F*_1,100_=11.85, *P*<.01; β=.07, SE 0.02).

^a^Study reported twice because it described the impact of both educational level and other skill-based proxies for health literacy on trust in online health information.

#### Use of Evaluation Criteria

Five studies (14%) investigated the last outcome of interest, namely people’s use of evaluation criteria for online health information (Table 5 [42,49-51,60]).

Only the study by Mackert et al [60] was about health literacy. This study, conducted in a low health literate-only sample, showed that low health literates used position in search results, quality of pictures, celebrity endorsement, and website authorship as criteria to evaluate online health information. With regard to website authorship, the study showed that almost all participants lacked trust in government or religious authorities as sources of online health information, whereas university researchers were generally considered trusted information providers.

Reliance on website authorship as an evaluation criterion was also found in one of the studies investigating the role of educational level. In his study, Dutta-Bergman [49] showed that educational level was positively associated with trust in information coming from medical universities and federal institutions and negatively associated with trust in information from health insurance companies. No differences related to education were found with regard to trust in online health information coming from the local doctor. Bates et al [42] showed that there was no consistent relationship between educational level and using readability of websites as a criterion to evaluate website quality. This finding suggests that ease of reading is not widely used as a criterion to evaluate the quality of the websites. Gauld and Williams [51] found that respondents with higher educational level were more likely to check credentials (eg, name or qualifications of the author) when evaluating health websites.

With regard to the role played by other skills-based proxies, Feufel and Stahl [50] showed in their study among skilled and less-skilled people that consistency with search intentions was used as a criterion to evaluate online health information. However, consistency referred to different things in the two groups: for the majority of less-skilled participants, this meant that a website confirmed a priori opinions, while for the majority of skilled participants, this meant that a website yielded the information that was searched for.

**Table 5:** Outcome 4: Use of evaluation criteria.

Author(s), date	Predictor	Evaluation criteria	Result
Mackert et al, 2009 [60]	Low health literacy (S-TOFHLA) only sample	Heuristics: Website position in search results; picture quality; celebrity endorsement; website authorship	Participants use heuristics to evaluate online health information quality: position in search results; quality of pictures; celebrity endorsement. Almost-universal lack of trust in the government and in religious figures as sources of online health information. University researchers are trusted sources as information providers.
Bates et al, 2007 [42]	Educational level	Website readability	No consistent relationship between educational level and using readability of websites as a criterion to evaluate website quality.
Dutta-Bergman, 2003 [49]	Educational level	Website authorship	Education does not impact trust on online health information from the local doctor. Trust in the local hospitals (*t=*3.83, *P*<.001) and in health insurance companies (*t*=1.90, *P*=.05) as sources of online health information is negatively associated with education. Individuals with higher trust in medical universities (*t*=11.83, *P*<.001) and federal sources (*t*=7.45, *P*<.001) are more educated than their counterparts.
Gauld & Williams, 2009 [51]	Educational level	Website credentials	Lower educational level decreases the likelihood to check credentials of health websites.
Feufel & Stahl, 2012 [50]	Other skills-based proxies for health literacy – Skilled (˃30 years of age, had a higher level of education, and were more experienced using the Web) vs less-skilled (≥50 years of age)	Consistency with search intentions	Overall, online health information is trusted if consistent with search intentions—among less-skilled participants this means if a website confirms a priori opinions (21/30, 70%) or yields search contents (9/30, 30%), and among skilled participants if a website confirms a priori opinions (4/28, 14%) or yields search contents (24/28, 86%).

##  Discussion

### Principal Findings

People’s health literacy is deemed to play an important role in the context of online health information seeking because according to most definitions it includes the ability to evaluate health information from different sources [12-19]. One of the main aims of this review was to identify and systematically summarize existing literature in order to collect evidence on the effect of low health literacy on the evaluation of online health information. The review provided us with indications of an overall (positive) association between health literacy (or one of its skills-based proxies) and both people’s ability to evaluate online health information (RQ1) and trust in the Internet as a source of health information (RQ3). On the other hand, evidence on the association between health literacy and both perceived quality of online health information (RQ2) and people’s use of evaluation criteria for online health information (RQ4) was inconsistent.

The limited number and the heterogeneity of studies using health literacy as a predictor makes it hard to get a clear picture of how people’s health literacy levels impact the evaluation of online health information. However, the included studies give us some indications that low health literacy may have a negative impact. Indeed, low health literate individuals use evaluation criteria that do not correspond to the well-established quality criteria [9]. To illustrate, one study reports that they do not trust online health information from the government or that they use the position of a website in search results or the quality of images to evaluate the quality of online health information [60], whereas evidence shows that information provided from institutional sources is usually accurate [80,81] and that position in search results and image quality are not among the criteria that should be used to judge the quality of a website [9]. This could at least partly explain why, as reported in another study [43], low health literate respondents, compared to their high health literate counterparts, have been shown to give higher-quality ratings to low-quality websites, and lower ratings to high-quality websites. At the same time, it has also been found that low health literacy is correlated with lower eHEALS scores (which include people’s perceived ability to evaluate online health information). Confidence in one’s own information skills has been shown to be positively related to information use [82].

What was observed in the few studies about health literacy was mostly confirmed in the higher amount of studies investigating differences in the evaluation of online health information related to educational level or other health literacy-related skills. Although we recognize that the diversity of measures used and the reliance on dichotomous measures of educational level may limit the accuracy of the results [40], this gives us more confidence in our findings. Included studies have shown that, overall, individuals with lower educational levels have worse actual and self-rated skills to evaluate the quality of online health information and lower trust in online health information compared to their more educated counterparts. Regarding perceived quality of online health information or people’s use of evaluation criteria, the limited number of studies and the diversity of samples and measures, however, does not allow us to draw conclusions about the impact of educational level or other skills-based proxies of health literacy, leaving two of the main research questions of this study mainly unanswered.

Besides providing us with indications on the role played by people’s health literacy in their evaluation of online health information, our review also highlighted some important gaps and limitations of research in this field. The main gap identified is probably the fact that, despite the undeniable theoretical importance of health literacy for online health information seeking, only four studies have specifically investigated the association between health literacy and the evaluation of online health information. Moreover, one of these studies was conducted in a low health literacy-only sample [60], not allowing comparisons across different health literacy levels. All the other studies in our review compared people with different educational levels or different levels of other literacy-related skills.

A second important limitation of current research highlighted by the review is the lack of shared definitions and measures. This has been shown to be the case not only for health literacy but also for other more commonly used predictors such as educational level, making comparisons across studies and summaries of existing evidence almost impossible. Additionally, only a few studies measured actual online health information evaluation skills, asking their participants to perform actual evaluation tasks. All the other studies relied on the participants’ self-rated ability. As shown in the study by van der Vaart et al [83], who compared their respondents’ eHEALS scores with an Internet performance test, self-rated ability does not seem to adequately capture people’s actual skills. Further research efforts should thus be devoted to the development and validation of a shared measure of online health information evaluation skills that is better able to reflect people’s real ability in this context.

Last, among the studies investigating more than one outcome variable, no information was found on the relationship between them. For this reason, no conclusions can be drawn on the interplay among the different evaluative dimensions.

### Limitations

This review has several limitations. The first limitation consists of the limited number of studies using health literacy as a predictor. Despite the fact that education has been shown to be related to (and it is often used as a proxy for) health literacy (eg, see [84]), this fact limits the extent to which our results can be generalized. A second important limitation of this review is the fact that all the included studies were non-interventional, with the consequence that the overall quality of the evidence has to be considered low [78]. Although causality is not an issue in this context (information evaluation cannot influence people’s literacy level), the uncontrolled nature of cross-sectional studies might fail to account for alternative explanations for the phenomenon under investigation [85]. This adds to the limited generalizability of the findings of this review. Finally, due to the heterogeneity of samples and outcome measures, it was not possible to conduct a meta-synthesis, with the result that no quantitative summary of the evidence could be provided.

### Conclusions and Future Directions

Despite its limitations, this systematic review on the role of health literacy in the evaluation of online health information has provided us with important insights on this topic. These insights have allowed us to draw some preliminary conclusions and, most importantly, have highlighted the main outcomes and limitations of current research in this relatively new and unexplored field.

From a research perspective, our findings are to be considered an indication of the fact that health literacy indeed plays a role in the evaluation of online health information and thus this topic is worth more scholarly attention. Based on the results of this review, the future research agenda in this field should include (1) a specific focus on health literacy, (2) more attention to the identification of the different criteria people use to evaluate online health information, (3) the development of shared definitions and measures for the most commonly used outcomes in the field of evaluation of online health information, and (4) the assessment of the relationship between the different evaluative dimensions and the role played by health literacy in shaping their interplay. Only by first addressing these research gaps will it be possible, in line with what has been called for in a recent Cochrane review [86], to develop high-quality interventions to enhance low health literacy individuals’ ability to appraise online health information as well as to develop well-designed randomized controlled trials to investigate their effects. A better understanding of how people appraise online health information is also crucial in view of the investigation of the impact of their information evaluation ability on their interactions with health care providers and ultimately on health outcomes.

From a practice perspective, the results of this review should be sufficient to urge public health officials and health care providers to start devoting particular attention to the online health information seeking behavior of low health literate citizens and provide them with targeted advice on criteria to correctly assess the quality of the information they find online.

## Multimedia Appendix 1

Search strategy.

## Abbreviations

CINAHL: Cumulative Index to Nursing and Allied Health Literature
eHEALS: eHealth Literacy Scale
HINTS: Health Information National Trends Survey
MeSH: Medical Subject Headings
S-TOFHLA: Short Test of Functional Health Literacy in Adults
TOFHLA: Test of Functional Health Literacy in Adults
