# Supplementary material for: Low Health Literacy and Evaluation of Online Health Information: A Systematic Review of the Literature
Source: J Med Internet Res. 2015 May 7;17(5):e112. doi: 10.2196/jmir.4018 (PMC4468598; doi:10.2196/jmir.4018)
Supplement: Supplementary file 1 [file jmir_v17i5e112_app1.pdf]

Search Strategy

Databases

PsycINFO	3.490 hits (January 24, 2014)
Medline	6.655 hits (January 24, 2014)
Web of Science	3.979 hits (January 24, 2014)
CINAHL	2.308 hits (January 24, 2014)
Communication & Mass Media Complete	1.075 hits (January 24, 2014)

PsycINFO

#1 Online information
computer assisted therapy/ OR online therapy/ OR telemedicine/ OR telemedicine.ti,ab,id. OR websites/ OR internet usage/ OR internet/ OR computer mediated communication/ OR
computer searching/ OR computer usage/ OR computer applications/ OR electronic communication/ OR (health adj3 website*).ti,ab,id. OR (health adj3 web).ti,ab,id. OR (health adj3 online).ti,ab,id. OR (health adj3 on-line).ti,ab,id. OR (health adj3 digital).ti,ab,id. OR
(health adj3 electronic*).ti,ab,id. OR (health adj3 internet).ti,ab,id. OR (health adj3 computer*).ti,ab,id. OR (health adj3 communication).ti,ab,id. OR (health adj3 information).ti,ab,id. OR information/ OR communication/ OR information seeking/ OR health care seeking behavior/ OR (health adj1 seeking behavio?r).ti,ab,id.
Results: 75.432 (January 24, 2014)

#2 Health
health/ OR health*.ti,ab,id.
Results: 437.609 (January 24, 2014)

#3 Evaluation
believab*.ti,ab,id. OR plausib*.ti,ab,id. OR assess*.ti,ab,id. OR apprais*.ti,ab,id. OR credibility/ OR credib*.ti,ab,id. OR "Trust (Social Behavior)"/ OR trust*.ti,ab,id. OR

truth/ OR
truth*.ti,ab,id. OR evaluation/ OR evaluation Criteria/ OR evaluat*.ti,ab,id. OR judgment/ OR
judg*.ti,ab,id. OR choice behavior/ OR choice behavio?r.ti,ab,id. OR quality.ti,ab,id. OR accura*.ti,ab,id.
Results: 1.030.219 (January 24, 2014)

#4 SES or LITERACY
educational attainment level/ OR Educational background/ OR Educat*.ti,ab,id. OR Employment Status/ OR Employment history/ OR Employ*.ti,ab,id. OR Unemployment/ OR Unemploy*.ti,ab,id. OR Job security/ OR Job*.ti,ab,id. OR Income Level/ OR "Income (Economic)"/ OR Income*.ti,ab,id. OR Social class/ OR Social class*.ti,ab,id. OR Minority Groups/ OR Minorit*.ti,ab,id. OR "Racial and Ethnic Groups"/ OR Racial group*.ti,ab,id. OR Ethnic group*.ti,ab,id. OR Disadvantaged/ OR disadvantaged.ti,ab,id. OR Health Disparities/ OR (health adj1 disparities).ti,ab,id. OR health care utilization/ OR health care utilization.ti,ab,id. OR poverty/ OR poverty.ti,ab,id. OR Socioeconomic Status/ OR Socioeconomic class attitudes/ OR socioeconomic.ti,ab,id. OR SES.ti,ab,id. OR underserved*.ti,ab,id. OR comprehension/ OR comprehension.ti,ab,id. OR number comprehension/ OR reading comprehension/ OR Reading Skills/ OR Reading Ability/ OR Reading achievement/ OR Reading development/ OR literacy/ OR literacy.ti,ab,id. OR information literacy/ OR reading/ OR reading.ti,ab,id. OR mathematical ability/ OR numeracy.ti,ab,id. OR numerical ability.ti,ab,id. OR health literacy/ OR health literacy.ti,ab,id. OR health education/ OR health education.ti,ab,id. OR health knowledge/ OR health knowledge.ti,ab,id.
Results: 740.507 (January 24, 2014)

1 AND 2 AND 3 AND 4: 3.666 (January 24, 2014)
1 AND 2 AND 3 AND 4 [NOT "0200 book" or "0240 authored book" or "0280 edited book"]: 3.490 (January 24, 2014)
